# Epipharyngeal Abrasive Therapy (EAT) Field Theory: A Clinical Framework for EAT in Modulating Psycho-Neuro-Endocrino-Immune (PNEI) Dynamics

**DOI:** 10.7759/cureus.93254

**Published:** 2025-09-26

**Authors:** Ito Hirobumi

**Affiliations:** 1 Otolaryngology, Ito Ear, Nose, and Throat (ENT) Clinic, Funabashi, JPN

**Keywords:** autonomic nervous system/physiology, eat field theory (eft), epipharyngeal abrasive therapy (eat), nasopharyngitis/chronic, nasopharynx/therapy, neuroimmunomodulation, neuroplasticity/physiology, polyvagal theory, psycho-neuro-endocrino-immunology, vagus nerve/physiology

## Abstract

Chronic epipharyngitis/chronic nasopharyngitis has traditionally been understood as a localized inflammatory disease, but clinically, it often presents with systemic symptoms that span the nervous, immune, and endocrine systems, such as headaches, fatigue, low-grade fever, impaired concentration, orthostatic dysregulation, joint pain, anxiety, and sleep disorders. Epipharyngeal abrasive therapy (EAT) is a treatment that applies chemical and physical stimulation to the mucous membranes of the epipharynx/nasopharynx using a cotton swab soaked in a 1% zinc chloride solution. Recently, there have been increasing reports of its efficacy in controlling chronic inflammation and improving multisystem symptoms. Regarding the mechanism of action of EAT, autonomic nervous system stimulation, immune system stimulation, and endocrine system stimulation have been reported; however, many aspects of the mechanism of action remain unclear.

A review of the existing research literature shows that EAT stimulates the autonomic nervous system through changes in parasympathetic activity mediated by the trigeminal-vagus reflex and has an immediate effect of inducing inflammatory reflexes. In addition, as a long-term effect, the autonomic nervous system balance is reestablished due to changes in the plasticity of the reflex pathway, chronic inflammation is controlled, and the immune response is normalized. Furthermore, continuous EAT stimulation influences the immune and endocrine systems, leading to the resynchronization of the body's homeostasis network.

This article systematizes the mechanisms by which EAT exerts its multi-systemic therapeutic effects on the nervous, immune, and endocrine systems, redefines EAT from a conventional local treatment to a systemic treatment, and presents the new framework of EAT field theory. This article is presented as a narrative and conceptual review rather than a systematic review, aiming to synthesize the clinical findings and theoretical perspectives into the proposed EAT field theory.

## Introduction and background

In the 1960s, Japanese otolaryngologist Shinsaku Horiguchi proposed the concept of nasopharyngitis and introduced a classification system that divided the condition into acute and chronic types. He devised the “B-spot therapy” as a treatment method [1], which was later developed by Tanaka A. into epipharyngeal abrasive therapy (EAT) [2].

In Japan, the terms “epipharynx,” “epipharyngitis,” and “EAT” have been used for a long time, but anatomically, the epipharynx is synonymous with the nasopharynx. In English-speaking countries, the term "nasopharynx" is generally used, and “epipharynx” is mainly limited to the fields of anatomy and zoology. The abbreviation EAT (epipharyngeal abrasive therapy) is also used in English-speaking countries to refer to epipharyngeal abrasive therapy, but in the future, the abbreviation NAT (nasopharyngeal abrasive therapy) may also be used. Standardization and clarification of terminology is an important issue in international medical communication. This paper aims to introduce EAT to English-speaking countries, so that it includes both the terms “epipharynx” and “nasopharynx” [3].

EAT is a treatment for chronic epipharyngitis/chronic nasopharyngitis that involves applying chemical and physical abrasive stimulation to the epipharyngeal/nasopharyngeal mucosa using a cotton swab soaked in 1% zinc chloride solution [1,2]. This treatment is performed with the aim of achieving local anti-inflammatory effects and mucosal stimulation. Traditionally, chronic nasopharyngitis has been recognized as a localized inflammatory disease; however, in clinical practice, there are numerous cases where patients present with systemic and diverse symptoms beyond localized symptoms. Typical symptoms include headaches, fatigue, low-grade fever, impaired concentration, orthostatic dysregulation, joint pain, anxiety, and sleep disorders, which constitute a syndrome-like presentation spanning the nervous, immune, and endocrine systems. The cause is often unclear, and the condition is frequently misdiagnosed as psychosomatic or functional in nature [4].

In recent years, with the expansion of clinical applications of EAT, cases have been reported in which multisystemic symptoms such as autonomic dysfunction [5], chronic fatigue [6,7], and post-COVID-19 syndrome (long COVID) [8-10] have improved simultaneously and transversally. These findings suggest that EAT may have a reintegrative effect on the body's physiological systems, rather than being merely a local treatment for chronic nasopharyngitis.

Hotta et al. proposed the epipharynx-limbic system axis interaction based on the clinical fact that chronic nasopharyngitis worsens due to stress, demonstrating the close relationship between the fields of otolaryngology and neurology [4]. However, at present, the specific mechanisms by which EAT affects the autonomic nervous system, immune system, and endocrine system, as well as their interactions, remain poorly understood. The authors hypothesize that the nasopharynx functions as an information hub connecting these multiple physiological systems and may play a crucial role in maintaining the body's homeostasis.

The nasopharynx is not merely a localized tissue but functions as a “field” that regulates the interrelationships between the autonomic nervous system, immune system, and endocrine system. When this field is impaired by chronic inflammation or other factors, the harmony between these systems is disrupted, leading to a variety of systemic symptoms. In other words, chronic nasopharyngitis should be understood not as a localized lesion but as a pathological condition resulting from the breakdown of systemic interconnections throughout the body. EAT is a treatment that directly acts on this “relational field” and has the potential to reintegrate and rebalance the disrupted system connections. The author has named this integrative framework the EAT field theory. In this review, the term “relational field” is operationally defined as the functional hub in which the autonomic nervous system, immune system, and endocrine system dynamically interact and co-regulate. Disturbances in this field result in multisystemic symptoms such as chronic fatigue, orthostatic intolerance, and cognitive dysfunction. Clinically, for example, improvements in heart rate variability in patients with myalgic encephalomyelitis/chronic fatigue syndrome (ME/CFS) or reduction of urinary protein in IgA nephropathy after EAT illustrate how repairing the relational field translates into measurable systemic outcomes. This theory redefines chronic nasopharyngitis from a local lesion model to a relational disruption model and positions EAT as “field repair medicine.”

## Review

Effects on the nervous system

The nasopharyngeal mucosa is innervated by the second branch of the trigeminal nerve (V2), the vagus nerve, and branches of the glossopharyngeal nerve. EAT stimulation activates both the sympathetic and parasympathetic nervous systems via these afferent pathways, inducing the EAT reflex. This reflex is thought to promote the reset of autonomic nervous activity and function as a trigger for neuromodulation [11]. Details of the autonomic reflex will be explained in the section “Autonomic Reflexes” and subsequent sections.

Effects on the immune system

The nasopharynx contains nasopharynx-associated lymphoid tissue (NALT), which serves as the front line of mucosal immunity, responsible for antigen presentation and cytokine production. In chronic nasopharyngitis, inflammatory cytokines such as tumor necrosis factor alpha (TNF-α), interleukin 6 (IL-6), and IL-1β are persistently elevated in NALT, contributing to systemic fatigue, low-grade fever, and immune hypersensitivity.

Nishi et al. conducted a comparative cross-sectional study of EAT for the treatment of chronic nasopharyngeal inflammation and reported that IL-6 mRNA was significantly reduced (p=0.0015) in the EAT group and that TNF-α was also suppressed [12,13]. Mogitate performed EAT for chronic nasopharyngitis and compared CD4+ and symptom Visual Analog Scores (VAS) before and after treatment. They reported that after three months of repeated EAT treatment, CD4+ cells significantly decreased (p=0.01) and VAS also significantly decreased (p<0.01) [14]. When EAT induces microscopic damage to the surface layer of the nasopharynx mucosa, it promotes remodeling of local inflammatory cells, readjusts the dedifferentiation and redifferentiation of immune cells, and calms excessive expression of inflammatory cytokines, which may result in immune re-education.

EAT has been reported to alleviate symptoms of autoimmune diseases such as IgA nephropathy. Mogitate reported in a retrospective study that weekly EAT for 12 weeks or more resulted in a reduction in urine protein (p<0.001) and negative urine occult blood (p=0.003). EAT was associated with a shortened hematuria period (p=0.047) and earlier improvement (p=0.041) [15]. EAT may be involved in the reconstruction of the homeostasis of the innate immune control mechanism.

Effects on the endocrine system

Endocrine signaling in the epipharynx affects food intake, satiety, and systemic metabolic control, playing an important role in maintaining energy homeostasis [16]. Since EAT treatment alleviates stress-related symptoms such as headaches, fatigue, low-grade fever, and sleep disorders, it is considered to influence the endocrine system. Some of the stimulating effects of EAT act on the hypothalamic-pituitary-adrenal (HPA) axis, promoting cortisol secretion [17] and normalizing diurnal cortisol fluctuations [18]. Additionally, it acts on the sympathetic-adrenal medullary (SAM) system to enhance salivary amylase activity and increase stress responsiveness [19].

EAT is thought to increase stress responsiveness because it affects changes in biomarkers of the endocrine stress axis. EAT contributes to maintaining homeostasis of the secretory system and normalizing stress responses.

Autonomic reflexes

The author conducted research focusing on the autonomic nervous system stimulation effects of EAT in order to elucidate its mechanism of action. The results revealed that EAT induces autonomic reflexes, and that the reflex patterns vary depending on the stimulation method, stimulation site, and timing. Additionally, immediate effects [20] and long-term effects [21] were observed, each with distinct mechanisms of action.

Immediate effect

First, when the posterior wall of the nasopharynx and the area near the canopy are stimulated by intranasal EAT, the parasympathetic nervous system is activated, inducing a vagal reflex (parasympathetic reflex). Clinically, this is observed as a decrease in heart rate and blood pressure. Next, when the entire nasopharynx is stimulated by oral EAT, the pharyngeal reflex is triggered, first activating the sympathetic nervous system, resulting in increased heart rate, increased alertness, and increased salivary amylase activity (with gender differences). Subsequently, with a time lag, the parasympathetic nervous system is activated, and the parasympathetic reflex appears. Between sympathetic and parasympathetic nervous activity, reflex compensation occurs. This is a mechanism by which the nervous system immediately responds to sudden changes in blood pressure, oxygen concentration, and body temperature to maintain homeostasis. The author defines these responses as immediate effects [19,20].

Long-term effect

**Figure 1:**
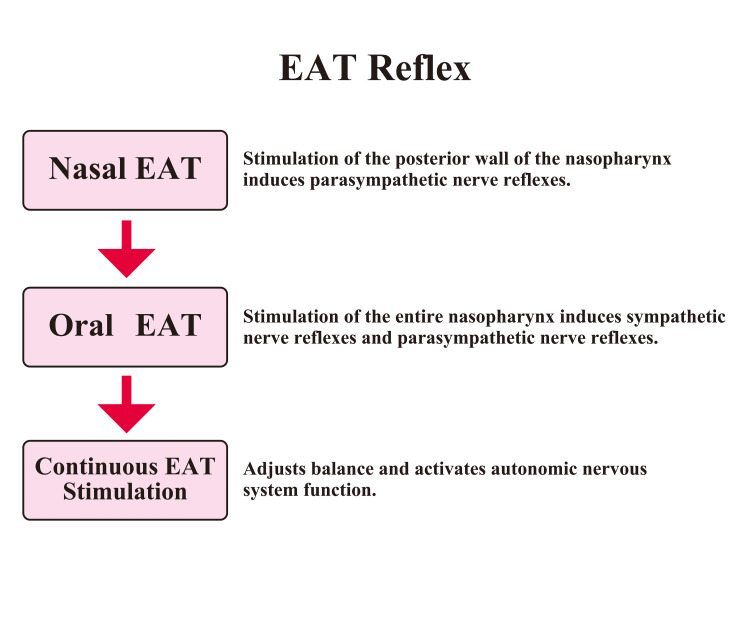
EAT Reflex The epipharyngeal abrasive therapy (EAT) reflex has both immediate and long-term effects. When the posterior wall of the epipharynx and the area near the epipharyngeal canopy are stimulated via nasal EAT, the vagus nerve reflex (parasympathetic reflex) is induced. When the entire epipharynx is stimulated via oral EAT, the pharyngeal reflex is induced. Initially, the sympathetic nervous system is activated, followed by the parasympathetic nervous system with a time lag. Reflex compensation occurs between sympathetic and parasympathetic nerve activity. When EAT is continuously applied, parasympathetic nerve activity is temporarily inhibited, and sympathetic nerve activity is activated. The sympathetic and parasympathetic nerves efficiently control homeostasis through reciprocal inhibition. Image Credit: Author

Continuous EAT treatment temporarily suppresses parasympathetic nerve activity and activates sympathetic nerve activity. At first glance, this seemingly counterintuitive change is part of the process of rebuilding autonomic nerve balance, whereby sympathetic nerve activation leads to enhanced parasympathetic nerve activity, ultimately forming a more stable balance. This process is an example of the allostatic response, a dynamic equilibrium control mechanism where the nervous, endocrine, and immune systems collaborate to establish a new equilibrium point. For instance, repeated EAT activates the baroreceptor reflex (BR), reducing the amplitude of blood pressure fluctuations. The sympathetic and parasympathetic nervous systems mutually inhibit each other, and through reciprocal inhibition, they efficiently control homeostasis. The author defines these reactions as long-term effects [19,21]. A summary of the EAT reflex is shown in Figure 1.

EAT reflex adjustment three-phase model

The autonomic nerve stimulation effect of EAT manifests in the following three stages. First, in the stimulation phase, mechanical friction stimulation calms local inflammation, and stimulation information is input from peripheral receptors to the nucleus tractus solitarius (NTS) via Aδ fibers and C fibers. Next, in the reflex phase, autonomic reflexes mediated through the medulla oblongata and hypothalamus are induced, resulting in immediate effects. Finally, in the remodeling phase, chronic inflammation is suppressed by continuous EAT, the autonomic reflex circuit is readjusted, and long-term effects are manifested. The authors have named this series of processes the “EAT reflex adjustment three-phase model” and propose it as the essential framework for the neural regulatory effects of EAT [5,11].

General neuroplasticity refers to learning and memory in the cerebral cortex and hippocampus, but in EAT, it is speculated that “neuroplasticity of reflex circuits” may be occurring. This neuroplasticity of reflex circuits may underpin the long-term effects of EAT. When reflex circuits such as the vagus nerve reflex or pressure receptor reflex are repeatedly stimulated, the structure and function of reflex centers such as NTS, rostral ventrolateral medulla (RVLM), dorsal motor nucleus of the vagus nerve (DMV), and spinal cord undergo changes and learning, thereby reconstructing autonomic nervous system functions independently of conscious control [22]. Clinically, while initial EAT stimulation may cause hypersensitive reflexes such as nausea, bradycardia, or coughing, we have observed cases where hypersensitive reactions subside with continued EAT treatment.

This suggests the possibility that the excitability of NTS may change depending on the input intensity and frequency of EAT, the possibility that long-term potentiation (LTP) may occur, in which synaptic transmission efficiency between neurons is strengthened over the long term due to the continuous repetition of neuronal activity in the NTS and DMV, or the possibility that long-term depression (LTD) may occur, in which synaptic transmission efficiency decreases over the long term. Additionally, the possibility that astrocytes and microglia surrounding the NTS may regulate excitatory environments, or that continuous stimulation may lower the threshold for reflexes, making them more likely to occur, or raise the threshold, making them less likely to occur, may be considered as reasons [23]. Repeated EAT adjusts the sensitivity of brainstem reflex pathways, reintegrating autonomic nervous system, immune system, and endocrine system responses.

**Figure 2:**
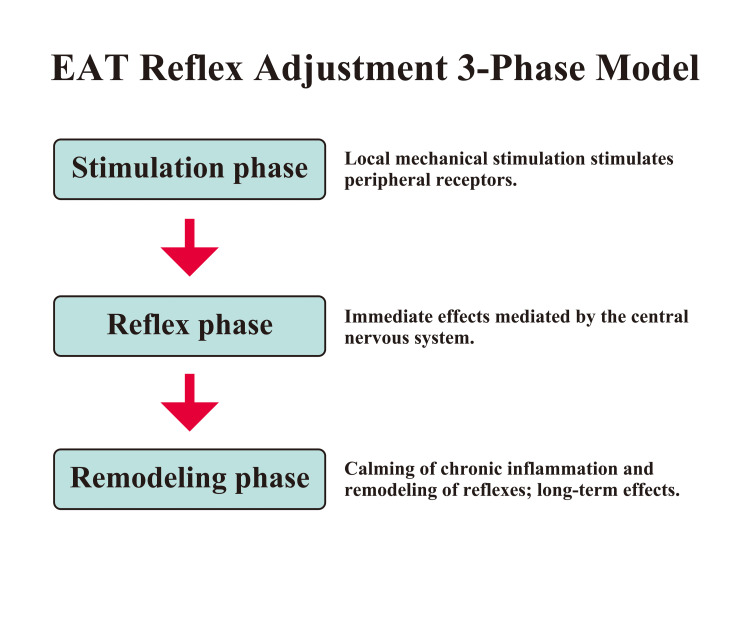
Epipharyngeal Abrasive Therapy (EAT) Reflex Adjustment Three-Phase Model The autonomic nervous system stimulation effect of EAT manifests in three phases. First, in the stimulation phase, mechanical friction stimulation calms local inflammation, and stimulation information is input from peripheral receptors. Next, in the reflex phase, autonomic nervous system reflexes are induced via the medulla oblongata and hypothalamus, and immediate effects are manifested. Finally, in the remodeling phase, chronic inflammation is suppressed by continuous EAT, the autonomic reflex circuit is readjusted, and temporal effects appear. Image Credit: Author

Ito conducted a study on cases of ME/CFS accompanied by chronic nasopharyngitis. Comparing 11 cases in the ME/CFS group and 18 cases in the non-ME/CFS group (total of 29 cases), the study found that the ME/CFS group exhibited significant changes in heart rate (p=0.000) and heart rate variability (p=0.012). EAT has been reported to potentially improve autonomic symptoms in ME/CFS [7,18]. Takezawa performed EAT on cases of postural orthostatic tachycardia syndrome (POTS) associated with long COVID and reported improvements in heart rate and blood pressure during the tilt test. EAT has been reported to potentially improve POTS [24]. EAT is considered to potentially restore function in chronic conditions such as autonomic dysfunction. EAT acts as a multi-level modulator of the nervous, immune, and endocrine systems. A schematic overview of the EAT reflex adjustment three-phase model is shown in Figure 2.

Vagus nerve inflammatory reflex and vagus nerve reflex

Both the vagus nerve reflex and the vagus nerve inflammatory reflex are important physiological reflexes involving the vagus nerve, but their roles differ.

The vagus nerve, as part of the parasympathetic nervous system, mediates the vagus nerve reflex, which regulates heart rate, respiration, and digestive functions. This reflex inhibits the sympathetic nervous system, lowering heart rate and blood pressure, and helps restore the body from a stressed state to a relaxed state, thereby contributing to the maintenance of homeostasis [23].

In addition, the vagus nerve has a vagus nerve inflammatory reflex that controls excessive inflammatory responses through interactions between the immune and endocrine systems [25,26]. When IL-1β and TNF-α levels rise in the periphery, the vagus nerve afferent pathway is stimulated, activating NTS-hypothalamus-paraventricular hypothalamic nucleus (PVN) pathway, activating the sympathetic nervous system and the hypothalamus-pituitary-adrenal (HPA) axis, thereby increasing cortisol secretion and suppressing the production of inflammatory cytokines.

Furthermore, activation of the vagus nerve efferent pathway and splenic sympathetic nerve pathway triggers the cholinergic anti-inflammatory pathway, where acetylcholine (ACh) acts on immune cells, particularly macrophages, to suppress the secretion of TNF-α and interleukins. This inhibition is mediated by α7 nicotinic acetylcholine receptors (α7nAChR) on immune cells [27]. Table 1 summarizes the vagus nerve inflammatory reflex and vagus nerve reflex.

**Table 1:** Comparison of vagus nerve inflammation reflex and vagus nerve reflex The vagus nerve is part of the parasympathetic nervous system and regulates heart rate, respiration, and digestive functions. The vagus nerve reflex contributes to maintaining homeostasis by restoring the body from a state of stress and leading it to a state of relaxation. The vagus nerve inflammation reflex controls excessive inflammatory responses through interactions between the immune and endocrine systems. This table was created by the author.

Features	Inflammatory Reflex	Vagus Nerve Reflex
Purpose	Regulation of inflammatory responses, suppression of excessive inflammatory responses to protect the body	Regulation of the autonomic nervous system, adjustment of heart rate, blood pressure, and digestive system activity
Mechanism	Acetylcholine binds to α7nAChR on macrophages, suppressing inflammatory cytokines such as TNF-α	The vagus nerve lowers the heart rate and regulates blood pressure via the parasympathetic nervous system.
Main role	Suppresses inflammatory responses and regulates excessive immune responses	Acts during relaxation and rest, regulating the cardiovascular system and breathing
Functions involved	Immune system, regulation of inflammatory responses	Regulation of heart rate, breathing, and digestive system
Representative conditions	Chronic inflammation, autoimmune diseases	Stress, tension, cardiovascular disease

Comparison of VNS and EAT

Vagus nerve stimulation (VNS) involves implanting stimulation electrodes in the left vagus nerve or applying electrical stimulation transcutaneously to act on the central nervous system, including NTS, locus coeruleus (LC), thalamus, and cortex. Additionally, it has immune-modulating effects through cholinergic anti-inflammatory reflexes. Clinically, it is applied to the treatment of drug-resistant epilepsy, treatment-resistant depression (TRD), migraine, cluster headache, inflammatory diseases, heart failure, autonomic dysfunction, POTS, ME/CFS, and other conditions [28].

VNS is an electrical stimulation therapy that involves neuroplasticity and brain function modification. It allows for frequency and voltage settings and has a wealth of research and evidence from overseas. There are two types: surgically implanted stimulation and non-invasive stimulation of the auricular vagus nerve through the skin. However, VNS does not necessarily always enhance parasympathetic activity. Under high-frequency, high-voltage conditions or with long-term stimulation using the same parameters, sympathetic dominance or desensitization may occur, leading to a decrease in parasympathetic activity. In states where the sympathetic nervous system is extremely activated (e.g., during the acute phase of POTS or ME/CFS), VNS-induced reflexes may temporarily enhance sympathetic nervous system activity [28]. Closed-loop VNS was developed with the aim of maximizing therapeutic effects. Unlike conventional open-loop VNS, which delivers stimulation at fixed parameters, closed-loop VNS adjusts stimulation based on the body's feedback. It is a system that immediately responds to dynamic changes in the autonomic nervous system and adjusts stimulation parameters (such as intensity and frequency) in real time to maximize therapeutic effects [29].

EAT is primarily a reflex stimulation therapy that uses physical friction stimulation of the nasopharyngeal mucosa, and it has local inflammatory control, immune modulation, and endocrine stimulation effects. The advantages of EAT include its low cost, simplicity, and the fact that it is an outpatient procedure that takes only a few minutes. However, it is technique-dependent and has issues with reproducibility. EAT is a treatment method that immediately induces vagus nerve reflexes, sympathetic nerve reflexes, BR, and endocrine system reflexes, among others. However, through these reactions, it exhibits neuro-regulatory therapeutic effects over time. In the future, immediate reflex responses are expected to be utilized as criteria for determining the appropriateness of EAT treatment and as indicators for evaluating inflammation, while changes in responses over time are anticipated to serve as indicators for assessing improvements in disease conditions [5,18]. Table 2 shows a comparison between VNS and EAT.

**Table 2:** Comparison of VNS and EAT VNS exerts its effects by electrically stimulating the central nervous system. It also participates in immune regulation via cholinergic anti-inflammatory reflexes. EAT is a reflex stimulation therapy using mechanical abrasive stimulation, which induces autonomic reflexes. It controls local inflammation and has immune regulatory and endocrine hormone stimulating effects. This table was created by the author. VNS: Vagus nerve stimulation; EAT: epipharyngeal abrasive therapy

Item	VNS	EAT
Purpose	Activates the vagus nerve through electrical stimulation to regulate the central and autonomic nervous systems	Stimulates the nasopharyngeal mucosa to reflexively regulate the autonomic nervous and immune systems
Stimulation Method	Direct electrical stimulation of the vagus nerve via surgical implantation or transcutaneous methods (auricular/neck)	Mechanical stimulation (abrasion of the nasopharyngeal mucosa using a cotton swab)
Main Stimulation Site	Left vagus nerve (neck, auricle, etc.)	Nasopharyngeal mucosa (reflex pathways of sympathetic and parasympathetic nerves)
Activated Nerve Fibers	Primarily C fibers (unmyelinated), partially Aδ fibers	Trigeminal nerve, C fibers (vagus nerve)
Main Functional Pathway	Vagus nerve → Nucleus tractus solitarius → Locus coeruleus → Cortex	Via trigeminal and vagus nerves → Nucleus tractus solitarius, thalamus, central reflex pathways
Characteristics of Action	Systemic regulation, immunosuppression, control of chronic inflammation	Local reflex regulation, short-term autonomic responses
Indications	Drug-resistant epilepsy, depression, chronic inflammatory diseases	Nasopharyngitis, myalgic encephalomyelitis/chronic fatigue syndrome (ME/CFS), autonomic dysfunction

Presentation of the EAT field theory

Systemic and multisystemic symptoms caused by chronic nasopharyngitis may be triggered by persistent neural stimulation activating the inflammatory circuit (IL-6 amplifier) [30] in the brainstem and hypothalamic vessels, thereby promoting the central invasion of autoreactive T cells via the gateway reflex [31]. The author proposes the hypothesis that this central inflammation disrupts the functions of the autonomic and endocrine centers, leading to symptoms such as chronic fatigue, insomnia, headaches, and orthostatic dysregulation [5]. Recent evidence further supports this systemic perspective. Yoon et al. demonstrated that the nasopharyngeal lymphatic plexus is a major hub for cerebrospinal fluid (CSF) drainage [32]. Chronic nasopharyngitis with mucosal edema may lead to lymphatic flow stasis, obstruction of CSF outflow, and elevated intracranial pressure, which in turn can impair hypothalamic-limbic system function. Within the framework of EAT field theory, this mechanism suggests that local inflammation in the nasopharynx may disrupt central homeostasis not only through neuroimmune reflexes but also via impaired CSF dynamics, and that EAT may help restore lymphatic-neuroendocrine balance by resolving mucosal inflammation. When local inflammation in the epipharynx is alleviated by EAT, inflammatory cytokines (IL-6, IL-1β, TNF-α) at the lesion site decrease, and abnormal excitation of submucosal sensory nerve endings also decreases. This disengages the trigger for the gateway reflex (inflammation circuit off, blood-brain barrier normalized), thereby alleviating central inflammation. As a result, it is speculated that recovery of autonomic nervous system function and endocrine function is promoted. The fact that systemic and multisystemic symptoms improve with EAT indirectly supports the existence of this neuro-immune feedback loop. However, direct evidence of the pathway from chronic nasopharyngeal inflammation to the gateway reflex in humans has not yet been reported. From the perspectives of physiology, neurology, immunology, and endocrinology, the nasopharynx has a unique structure and function as a hub connecting these multiple systems. When chronic inflammation continuously sends persistent noise to this intersection, the mutual adjustment functions of each system break down, causing systemic disruption. In other words, chronic nasopharyngitis is not limited to a local disease, but can be the starting point for disruption of the entire body's network.

**Figure 3:**
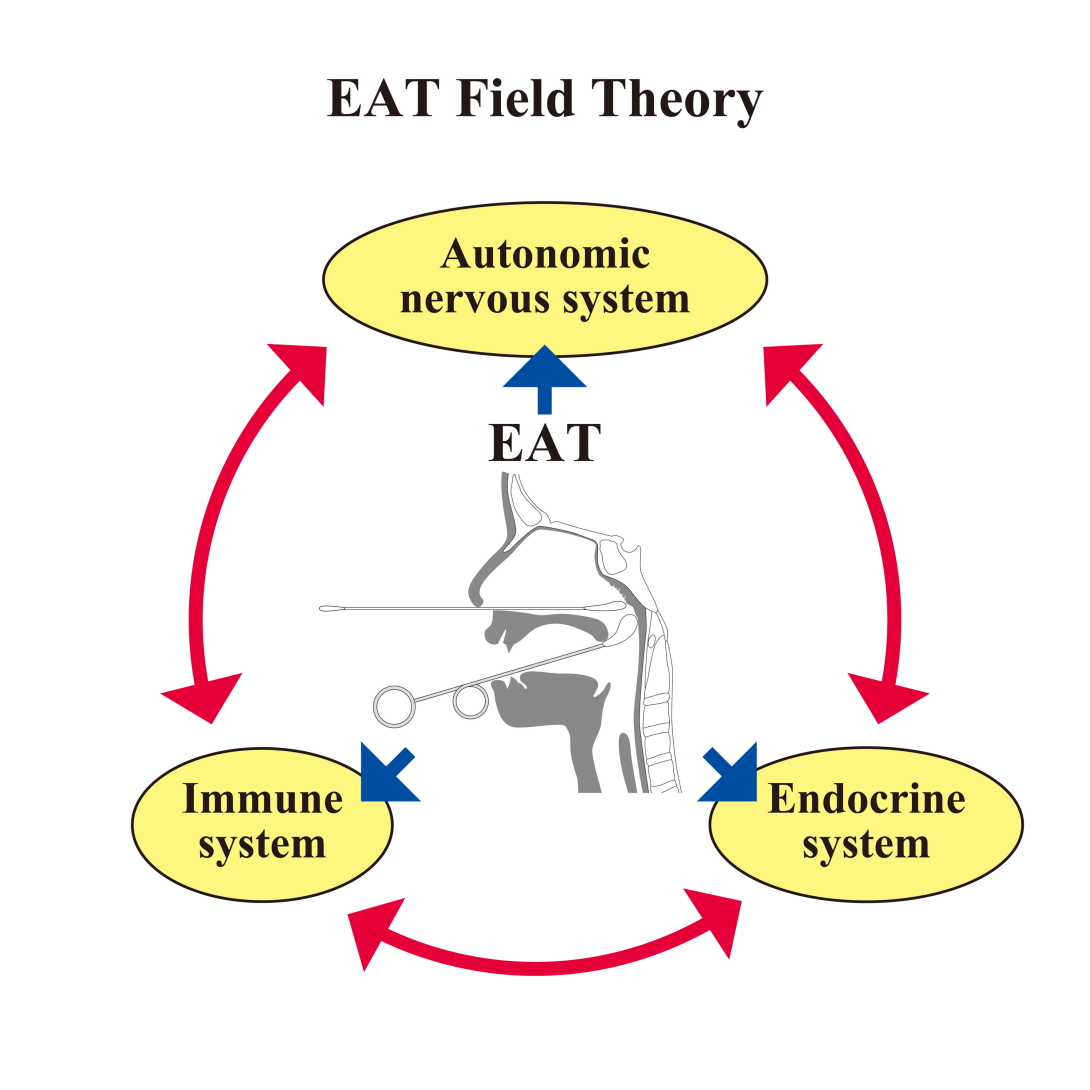
EAT Field Theory Epipharyngeal abrasive therapy (EAT) field theory redefines the causes of chronic epipharyngitis symptoms from a holistic perspective, considering not only the epipharynx itself but also disturbances in the autonomic nervous system, immune system, and endocrine system. EAT is a treatment that acts on the epipharynx, which is the hub of the relationships between these systems, to repair, adjust, and promote system reintegration; in other words, it is a treatment that repairs the field of relationships. Image Credit: Author

From this perspective, EAT is not simply a means of eliminating local inflammation; it is positioned as a stimulus that reintegrates the relationships between multiple physiological systems and resets structure and function across the board. EAT field theory is a hypothesis that comprehensively explains the theoretical basis of this systemic effect. Rather than attributing the symptoms of chronic nasopharyngitis solely to the local nasopharynx, EAT field theory redefines it as a systemic disease characterized by the disruption of the relational fields of the autonomic nervous system, immune system, endocrine system, and lymphatic system. EAT is positioned as a therapeutic approach to repair this systemic disruption. By redefining EAT from a holistic perspective of relational disruption, it goes beyond mere physical abrasion and can be considered a treatment that acts on, repairs, and adjusts the relationship hubs of the autonomic nervous system, immune system, and endocrine system, promoting system reintegration - in other words, a treatment that repairs the relational field. EAT field theory is a hypothesis that comprehensively explains this systemic effect and presents a new framework for "medicine that repairs the relational field." Figure 3 illustrates the EAT field theory.

The relationship between traditional Eastern medicine theory and EAT field theory

It should be noted that references to Eastern medical theory in this review are not intended to serve as direct scientific evidence but rather as conceptual analogies to help international readers contextualize the systemic framework of EAT field theory. The parallels with “qi, blood, and body fluids” or the five zang-six fu organ interactions are presented as heuristic models that resonate with modern PNEI theory, while remaining distinct from the biomedical evidence base.

Western medicine often views the body as a collection of parts and tends to attribute the cause of illness to a single organ or function. In contrast, EAT field theory adopts a traditional Eastern medical perspective, positing that symptoms arise from disruptions in the relationships between systems and that healing begins with the harmonization of these relationships. In traditional Eastern medicine, health is maintained through the harmonious circulation of “qi, blood, and body fluids” and the balanced interactions among the five zang organs and six fu organs. In particular, the concept of adjusting the overall balance at the “pre-disease” stage aligns well with modern medicine as a preventive intervention for chronic diseases [33]. EAT is a treatment that acts on the upper pharynx, a crossroads of bodily relationships, to restore harmony in the autonomic nervous system, immune system, and endocrine system. It is positioned as “field medicine” from a traditional Eastern medical perspective. EAT field theory resonates deeply with traditional Eastern medical theory in that it goes beyond local treatment to remove inflammation and promotes the reintegration of relational fields.

The relationship between polyvagal theory and EAT field theory

Polyvagal theory, proposed by Stephen Porges, is a theory that demonstrates that the autonomic nervous system has an evolutionary hierarchical structure. In particular, the ventral vagal nervous system mediates “social engagement” and “neuroception of safety.” According to this theory, disruptions in social relationships can negatively impact the nervous system, while repairing social relationships can enhance the nervous system's sense of safety and restore neural activity. Under chronic inflammation or prolonged stress, a sympathetic nervous system dominance persists, and the activation of the dorsal vagal nervous system can lead to freeze responses (fatigue, apathy, dissociation) [34]. EAT may activate the ventral vagal system through the vagal reflex, restore the social engagement system, and bring about a sense of safety and security [20]. This effect resonates deeply with the polyvagal theory, suggesting that EAT may help reconstruct the hierarchical balance between the sympathetic and parasympathetic nervous systems.

The relationship between PNEI theory and EAT field theory

Psycho-neuro-endocrino-immunology (PNEI) theory is a theory that posits that human homeostasis is maintained not by a single system, but by the complex interactions of psychological, neurological, endocrine, and immune networks [35]. PNEI encompasses psychology (emotions, cognition, stress, trauma), neurology (regulation by the central nervous system and autonomic nervous system), endocrinology (hormone secretion centered on the HPA axis), and immunology (immune response, Th1/Th2 balance, cytokines, antibodies, etc.). These systems are interconnected and possess bidirectional feedback mechanisms. Psychological stress alters immune responses through the autonomic nervous system and hormones, while inflammation and immune activity, in turn, influence emotions and cognition [36,37]. EAT may stimulate autonomic nervous system activity and, by extending to the immune and endocrine systems, potentially resynchronize the body's PNEI network. This extended effect is key to understanding the multifaceted efficacy of EAT, which is difficult to explain using traditional organ-specific causal models. The EAT field theory can be presented as a multidimensional treatment theory.

Future prospects and challenges

EAT field theory views the nasopharynx as a field of relationships and positions EAT as a medical treatment that repairs and maintains this field, restoring harmony between systems. The therapeutic effects of EAT are thought to be manifested not only by simply adjusting the structure of the epipharynx but also by reconstructing the epipharynx as a hub of physiological, neurological, immunological, and endocrinological relationships, thereby resynchronizing the disrupted networks within the body.

This theory is based on the author's clinical experience; however, the therapeutic effects of EAT exhibit diverse response patterns and significant individual variability. Therefore, further clarification of the physiological and neuroimmunological mechanisms underlying the multifaceted effects of EAT is necessary. In terms of clinical research, key challenges include accumulating case numbers, conducting randomized controlled trials (RCTs), and verifying reproducibility and generalizability through multicenter prospective studies [38]. Additionally, the evaluation of the severity and improvement of chronic nasopharyngitis should not rely solely on subjective scores but should incorporate objective scores and multi-system biomarkers (such as cortisol, salivary amylase activity, fractional exhaled nitric oxide (FeNO), and inflammatory cytokines) for quantitative assessment. The autonomic nervous system function evaluation using heart rate variability (HRV) analysis conducted by the authors is useful, but it has limitations when used alone and should be utilized as a composite indicator. Furthermore, the establishment of standardized evaluation methods and procedural guidelines for EAT is essential to promote its clinical adoption.

This review should be understood as a narrative and conceptual review rather than a systematic review. While the literature search included PubMed, CiNii, and Ichushi databases using terms such as “epipharyngitis,” “EAT,” and “autonomic reflex” (2017-2025), no formal PRISMA-based inclusion/exclusion criteria or risk-of-bias assessments were applied. The primary aim is to synthesize recent findings and integrate them into the proposed EAT field theory framework, rather than to quantitatively pool data through meta-analysis.

## Conclusions

As this review is primarily conceptual and narrative in scope, it does not include quantitative synthesis such as meta-analysis or meta-regression. No pooled statistical indicators (e.g., p-values, confidence intervals) are presented. Instead, individual studies are cited qualitatively to support the construction of the EAT field theory.

Autonomic reflexes induced by EAT have immediate and long-term effects. Continuous stimulation controls chronic inflammation and promotes normalization of autonomic reflexes and reconstruction of functional neuroplasticity.

EAT field theory redefines chronic nasopharyngitis not as a localized lesion, but from a holistic perspective that considers the disruption of the relationship between the autonomic nervous system, immune system, and endocrine system. EAT is a medical treatment that restores systemic harmony throughout the body by regulating the nasopharynx, which is the site of these relationships, and is positioned as the theoretical basis for future clinical research.
